# Analysis of the natural collapse course of non-traumatic osteonecrosis of the femoral head based on the matrix model

**DOI:** 10.1186/s13018-024-04587-1

**Published:** 2024-01-31

**Authors:** Rushun Zhao, Mengfei Wang, Yangquan Hao, Peng Xu, Chao Lu

**Affiliations:** 1 Department of Joint Surgery, Xi’an Hong Hui Hospital, Xi’an Jiaotong University Health Science Center, No.555 Youyi East Road, Nanshaomen, Xi’an, 710054 Shaanxi Province People’s Republic of China; 2 Shaanxi University of Traditional Chinese Medicine, Xi’an, 712046 Shaanxi Province People’s Republic of China

**Keywords:** Femoral head, Collapse, Matrix model, Risk factors, Prediction

## Abstract

**Background:**

There are many predictions about the progression of natural collapse course of osteonecrosis of the femoral head. Here, we aimed to combine the three classical prediction methods to explore the progression of the natural collapse course.

**Methods:**

This retrospective study included 127 patients admitted to our hospital from October 2016 to October 2017, in whom the femoral head had not collapsed. Logistic regression analysis was performed to determine the collapse risk factors, and Kaplan–Meier survival curves were used for femoral head survival analysis. The collapse rate of the femoral head was recorded within 5 years based on the matrix model. The specificity of the matrix model was analyzed using the receiver operating characteristic curve.

**Results:**

A total of 127 patients with a total of 202 hips were included in this study, and 98 hips collapsed during the follow-up period. Multivariate logistics regression analysis showed that the predictive ability of the matrix model was stronger than Association Research Circulation Osseous staging, Japanese Investigation Committee classification, and area (*P* < 0.05). Kaplan–Meier survival curve showed that the median survival time of femoral head in patients was 3 years. The result of the receiver operating characteristic curve analysis showed that the area under the curve (AUC) of the matrix model had better predictive value (AUC = 0.771, log-rank test: *P* < 0.001).

**Conclusion:**

We creatively combined the three classical prediction methods for evaluating the progression of the natural collapse course based on the matrix model and found that the higher the score of the matrix model, the higher the femoral head collapse rate. Specifically, the matrix model has a potential value in predicting femoral head collapse and guiding treatment selection.

## Introduction

Osteonecrosis of the femoral head is a common refractory disease in the field of orthopedics, characterized by damage to the trabecular structure or even collapse, and is more common in symptoms such as hip pain and limited mobility [1]. Osteonecrosis of the femoral head is divided into traumatic and non-traumatic osteonecrosis of the femoral head (NONFH), among which NONFH is mostly caused by glucocorticoids, alcohol, and other factors [2]. At present, the specific pathogenesis of osteonecrosis of the femoral head is not clear, and there are no targeted drugs for treatment [3]. According to previous reports, the number of patients with NONFH has exceeded eight million in China, and in recent years the morbidity has become associated with younger people [4]. Collapse of the femoral head is a critical point in the progression of NONFH, and when it occurs, it can adversely affect the normal function of the hip joint, eventually resulting in joint replacement [5]. Therefore, accurate prediction of the collapse risk can help to identify collapse early and prevent it through correct intervention [6].

First, we should know the natural collapse course of NONFH in patients who did not undergo surgical intervention and without collapse of femoral head; hence, in our study, most of the patients were collected from outpatient clinics instead of the inpatient departments [7]. Next, we must understand the stage of femoral head necrosis through imaging data that can predict the natural collapse progression of NONFH. Although there are many methods for predicting the collapse of osteonecrosis of the femoral head, they have not been applied to clinical practice, and the internationally recognized factors are mainly based on the staging of the Association Research Circulation Osseous (ARCO), Japanese Investigation Committee (JIC) classification, necrosis lesion location, and area [8–11]. However, the predictive power of these factors individually is poor. The matrix model is a commonly used thinking tool that comprehensively evaluates research objects through multidimensional independent influencing factors. For example, Dr. Yskert von Kölsch’s team used the SWOT matrix to analyze personalized medical strategies for aortic disease [12]. Therefore, we creatively proposed a matrix model that can comprehensively analyze the ARCO staging, JIC classification, and area that affect the collapse of femoral head, in order to predict the natural collapse process of NONFH. In summary, we conducted a retrospective study on outpatient patients without femoral head collapse and proposed a matrix model to predict the progression of the natural collapse process of NONFH.

## Materials and methods

### Case inclusion and exclusion criteria

Case inclusion criteria: (1) The femoral head has not collapsed; (2) patients with complete imaging data; (3) patients who have not undergone surgical intervention treatment.

Case exclusion criteria: (1) Patients whose imaging data cannot determine the staging or typing; (2) patients who have lost follow-up; (3) patients who had previously undergone hip-preserving surgery or other surgical treatments; (4) patients with severe diabetes and major cardiovascular diseases.

### Patient enrollment

This retrospective study conforms to the Declaration of Helsinki, revised in 2013, and was approved by the Medical Ethics Committee of the Honghui Hospital Affiliated to Xi’an Jiaotong University (approval number: 202212002). All patients gave informed consent and signed an informed consent form. In this study, we included 127 patients diagnosed with NONFH, where the femoral head had not collapsed, admitted to the Honghui Hospital Affiliated to Xi’an Jiaotong University from October 2016 to October 2017. Among them, there were 48 cases of steroid-induced avascular necrosis of the femoral head, 13 cases of alcohol-induced osteonecrosis of the femoral head, and 66 cases of idiopathic osteonecrosis of the femoral head, and idiopathic NONFH refers to the condition where the specific cause is unclear. Besides, there were 52 unilateral cases and 75 bilateral cases, and there were 86 male patients and 41 female patients. The mean age was (50.20 ± 12.76) years, and the mean body mass index (BMI) was 22.94 ± 2.54. All baseline data are shown in Table 1.

**Table 1:** Patient demographics

Demographic	
Patients (M/F)	127
Male	86
Female	41
Mean age (range), y	50.20 ± 12.76
BMI ($\bar{x}$ ± s, kg/m^2^)	22.94 ± 2.54
invasive hip	
Unilateral	52
Bilateral	75
etiology	
alcohol	13
Corticosteroids	48
idiopathic	66

All qualitative variables are presented as numbers except age and BMI, which are presented as mean ± standard deviation

*BMI* body mass index

### Methods

In this study, the height, weight, causative factors, and time of first diagnosis were determined by telephone follow-up, and the time of femoral head collapse and the current femoral head collapse were determined according to the imaging data, fed back by the patient's outpatient clinic or the Internet. During follow-up, patients without collapse of the femoral head occasionally experience symptoms such as pain and functional impairment, but the symptoms are mild and do not affect daily life. Collapse criteria**:** Collapse was considered by imaging data or total hip replacement.

### The matrix models

We scored ARCO I and II staging as 1 point and 2 points, respectively. If the area of osteonecrosis was less than 50%, it was scored as 1 point, and if greater than 50%, it was scored as 2 points. The JIC classification was scored as 1 point for the position involving the inner column (type A + B) and 2 points for the position involving the outer column (type C1 + C2), according to the different locations of the necrosis lesion. We finally multiplied the scores of the three to assess the progression of the natural collapse process of NONFH.

### Imaging materials

In terms of imaging data, two professionally trained orthopedic surgeons measured the imaging data separately, and any disputes were resolved through discussion and negotiation. X-rays and MRIs are used to assess the stage and type of osteonecrosis of the femoral head, and CT was used to assess the size of the necrotic area.

### Statistical analysis

SPSS 26.0 software (version 26.0; IBM, Armonk, New York, USA) was used for statistical analysis. The measurement data were expressed as $\bar{x}$ ± s (mean ± standard deviation), and the independent sample *t*-test was used for the comparison between groups that conformed to the normal distribution. The counting data were expressed as percentages, and the Chi-square test was used for comparison between groups. Multivariate logistic regression was used to analyze collapse risk factors, and Kaplan–Meier survival curves were used for femoral head survival analysis. Using the receiver operating characteristic curve, predict and evaluate the specificity of the matrix model. Statistical significance was set at *P* < 0.05.

## Results

A total of 127 patients with a total of 202 hips were included in this study, and 98 hips collapsed during the follow-up period, with a total collapse rate of 48.5%. There were no significant differences in the mean BMI (*P* = 0.725), sex (*P* = 0.426), incidence side (*P* = 0.473), and Etiology (*P* = 0.608)between two groups. It is worth noting that univariate analysis showed that there were statistically significant differences between the collapse group and the non-collapse group in terms of age (*P* = 0.006), ARCO staging, JIC classification, and area (*P* < 0.001). In terms of ARCO stages, ARCO stage I and II collapsed four hips (2.0%) and 94 hips (46.5%), respectively. In terms of JIC classification, type A, type B, type C1, and type C2 collapsed two hips (1.0%), 16 hips (7.9%), 48 hips (23.8%), and 32 hips (15.8%), respectively. In terms of necrotic area, 30 hips (14.9%), 40 hips (19.8%), and 28 hips (13.9%) were collapsed in < 30%, 30%–50% and > 50% area, respectively. All data are shown in Table 2.

**Table 2:** Univariate analysis of 202 hips between collapse and non-collapse groups

	Total	Collapse	Non-collapse	*P* value
	*n*	*n*/%	*n*/%	
Number of hips	202	98/48.5	104/51.5	
Age (x ± s)	52.48 ± 12.18	47.60 ± 12.57	0.006^a^**	
BMI (x ± s)	23.03 ± 2.76	22.90 ± 2.47	0.725^a^	
Sex				0.426^b^
Male	141	71/35.1	70/34.7	
Female	61	27/13.4	34/16.8	
Invasive hip				0.473^b^
Unilateral	52	23/11.4	29/14.4	
Bilateral	150	75/37.1	75/37.1	
Etiology				0.608^b^
Idiopathic	100	52/25.7	48/23.8	
Glucocorticoid	79	36/17.8	43/21.3	
Alcohol	23	10/5.0	13/6.4	
ARCO staging				0.000^b^***
Stage I	40	4/2.0	36/17.8	
Stage II	162	94/46.5	68/33.7	
JIC classification				0.000^b^***
Type A	19	2/1.0	17/8.4	
Type B	54	16/7.9	38/18.8	
Type C1	86	48/23.8	38/18.8	
Type C2	43	32/15.8	11/5.4	
Area				0.000^b^***
< 30%	91	30/14.9	61/30.2	
30–50%	77	40/19.8	39/19.3	
> 50%	34	28/13.9	4/2.0	

*ARCO* the Association Research Circulation Osseous, *JIC* Japanese Investigation Committee

*Significant difference (*P* < 0.05)

**Extremely marked difference (*P* < 0.01)

***Extremely marked difference (*P* < 0.001)

^a^Independent sample *t*-test

^b^Chi-square test

Next, we performed a univariate analysis of the matrix model, and the results showed that the collapse rates of patients with scores of 1, 2, 4, and 8 were 4.0%, 32.3%, 58.8%, and 90.0%, respectively, and the difference was statistically significant (*P* < 0.001). All data are shown in Table 3.

**Table 3:** Univariate analysis of the matrix model

	Total	Collapse	Non-collapse	*P* value
	*n*	*n*/%	*n*/%	
Number of hips	202	98/48.5	104/51.5	0.000^b^***
One point	25	1/4.0	24/96.0	
Two point	62	20/32.3	42/67.7	
Four point	85	50/58.8	35/41.2	
Eight point	30	27/90.0	3/10.0	

***Extremely marked difference (*P* < 0.001)

^b^Chi-square test

Subsequently, further multivariate analysis showed that there were statistically significant differences in age (P = 0.005, adjusted odds ratio [OR] = 1.041, 95% confidence interval [CI]: 1.012–1.070) and ARCO stage (*P* < 0.001, adjusted OR = 9.175, 95% CI: 2.939–28.645), while there was no statistically significant difference between type B(*P* = 0.321)and type C1(*P* = 0.054)in JIC classification, and there was a statistically significant difference in C2 type (P = 0.014, adjusted OR = 9.292, 95% CI: 1.582–54.583). There was no statistically significant difference in necrosis area in the 30%–50% interval (*P* = 0.869), while there was a statistically significant difference in the > 50% interval (*P* = 0.025, adjusted OR = 4.327, 95% CI: 1.203–15.559). In the matrix model, the adjusted OR of 2, 4, and 8 points were 13.991, 40.993, and 263.180, respectively, and all of them were statistically different (*P* < 0.05), indicating that the model was closely related to femoral head collapse (Table 4).

**Table 4:** Multivariate logistic regression analysis of risk factors

	B	SE	Wald	*P* value	OR	95% CI	
Age	0.040	0.014	7.979	0.005^c^**	1.041	1.012	1.070
ARCO staging							
Stage I					1		
Stage II	2.216	0.581	14.559	0.000 ^c^***	9.175	2.939	28.645
JIC classification							
Type A					1		
Type B	0.855	0.861	0.986	0.321^c^	2.35	0.435	12.699
Type C1	1.629	0.847	3.702	0.054^c^	5.099	0.97	26.799
Type C2	2.229	0.903	6.088	0.014^c^*	9.292	1.582	54.583
Area							
< 30%					1		
30–50%	−0.065	0.397	0.027	0.869^c^	0.937	0.43	2.040
> 50%	1.465	0.653	5.032	0.025^c^*	4.327	1.203	15.559
Matrix model							
One point					1		
Two point	2.638	1.067	6.115	0.013^c^*	13.991	1.729	113.237
Four point	3.713	1.056	12.377	0.000 ^c^***	40.993	5.179	324.470
Eight point	5.573	1.206	21.344	0.000 ^c^***	263.180	24.744	2799.68

*ARCO* the Association Research Circulation Osseous, *JIC* Japanese Investigation Committee, *OR* odds ratio, *CI* confidence interval

*Significant difference (*P* < 0.05)

**Extremely marked difference (*P* < 0.01)

***Extremely marked difference (*P* < 0.001)

^c^Multivariate logistic regression analysis

The number and rate of collapsed hips with points of 1, 2, 4, and 8 within 1 year were one hip (4.0%), 15 hips (24.2%), 31 hips (36.5%), and 12 hips (40.0%), respectively; the number and rate of collapsed hips within 3 years were one hip (4.0%), 18 hips (29.0%), 46 hips (54.1%), and 19 hips (63.3%), respectively; and the number and rate of collapsed hips within 5 years were one hip (4.0%), 20 hips (32.3%), 50 hips (58.8%), and 27 hips (90.0%), respectively. All the differences were significant (*P* < 0.01). The above data are shown in Table 5.

**Table 5:** The collapse rate of femoral head within 5 years based on the matrix model

	One point (*n* = 25)	Two point (*n* = 62)	Four point (*n* = 85)	Eight point (*n* = 30)	Statistical value	*P* value
	*n*/%	*n*/%	*n*/%	*n*/%		
1-year collapse	1/4.0	15/24.2	31/36.5	12/40.0	*χ*^2^ = 12.295	0.006**
3-year collapse	1/4.0	18/29.0	46/54.1	19/63.3	*χ*^2^ = 29.897	0.000***
5-year collapse	1/4.0	20/32.3	50/58.8	27/90.0	*χ*^2^ = 50.680	0.000***

**Extremely marked difference (*P* < 0.01)

***Extremely marked difference (*P* < 0.001)

**Fig. 1:**
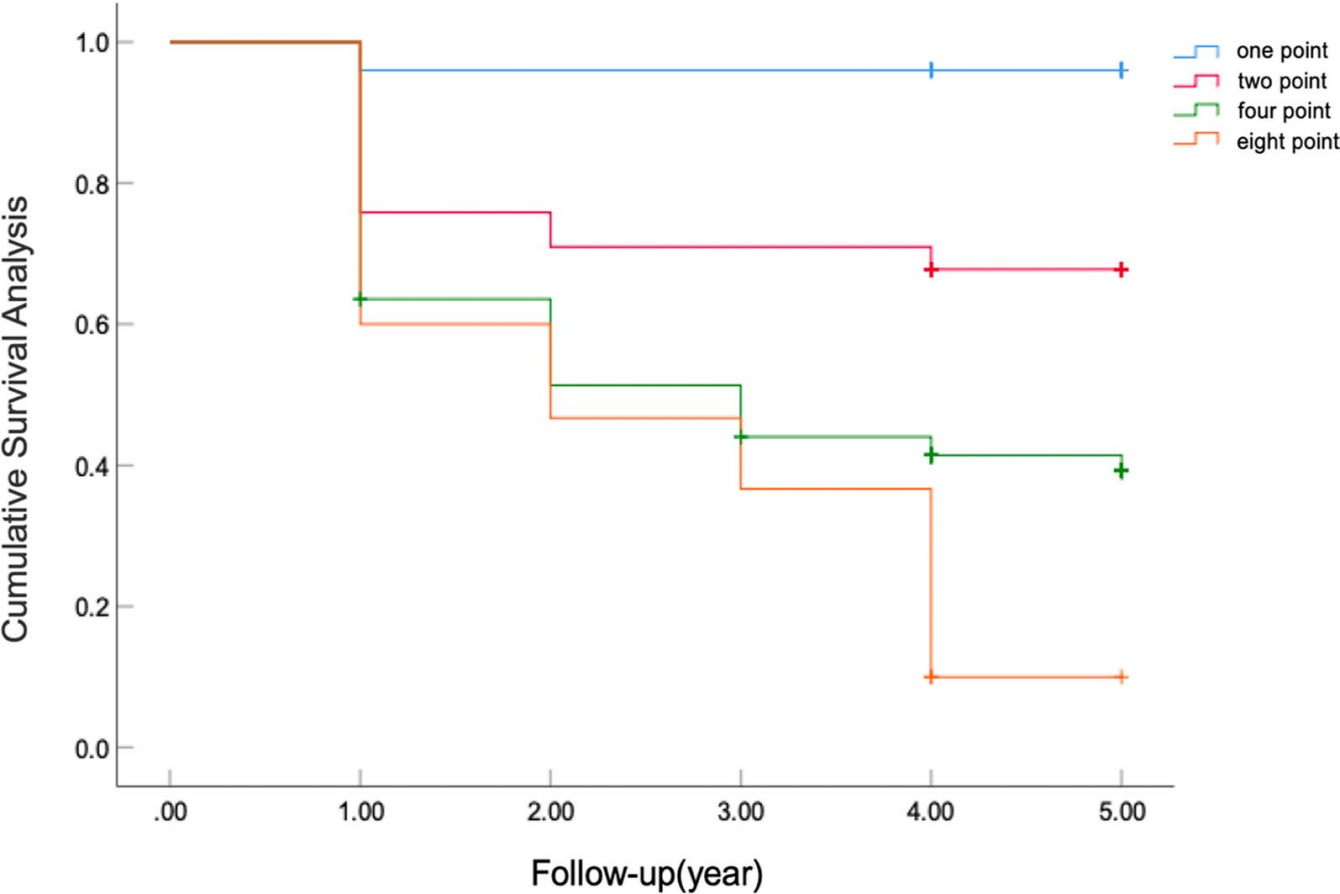
Kaplan–Meier survival curve of the matrix model

**Fig. 2:**
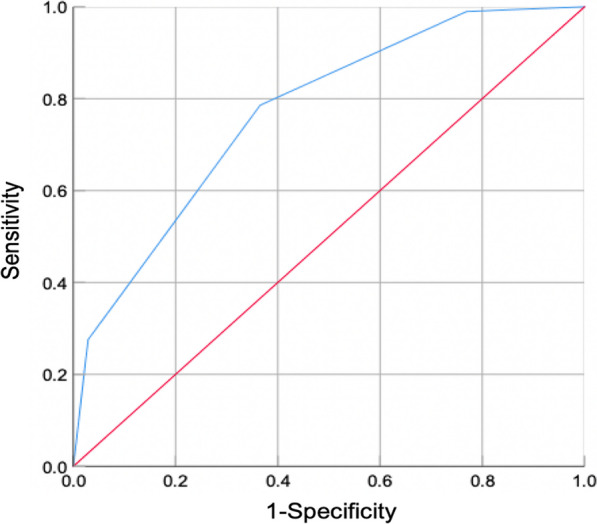
ROC curve analysis of the matrix model. ROC, receiver operating characteristic

The Kaplan–Meier survival curve showed that the median survival time of femoral head in patients was 3 years (95% CI: 3.176–3.672 years), and the non-collapse rates of femoral head within 1, 3 and 5 years were 70.8% (143/202), 58.4% (118/202) and 51.5% (104/202), respectively (Fig. 1). Statistical analysis of the femoral head survival of patients through the matrix model showed that the survival rate of femoral head was lower with the higher score of the matrix model, and the log-rank test showed that the difference in femoral head survival rate of patients with different scores was significant (*χ*^2^ = 45.725, *P* < 0.001). Receiver operating characteristic curve analysis showed that the area under the curve (AUC) of the matrix model had better predictive value (AUC, 0.771; 95% CI: 0.707–0.834), and the cut-off value is 3 point, at which point the sensitivity 0.786 in sensitivity and 0.635 in specificity and the difference was significant (*P* < 0.001) (Fig. 2). It shows that the prediction accuracy of this method is high and the method has practical value.

## Discussion

The early diagnosis and prognosis of NONFH have always been a research hotspot in orthopedics [13–15]. Many prediction models for femoral head necrosis and collapse have emerged in recent years. For example, Professor Wei He [16] proposed prediction of the prognosis of collapse for NONFH by measuring the anterior lateral angle of the femoral head, and Professor Liming Cheng [17] proposed that necrosis involved in the subchondral bone is more prone to collapse. However, these new predictive models have not been widely applied, and previous studies [18, 19] have shown that the collapse of the femoral head is closely related to staging, classification, and area size, especially the preservation of the lateral column, which plays an important role in the survival of the femoral head [20, 21]. However, relying on a new method to forecast the collapse of femoral head necrosis is not comprehensive, and we need to integrate multiple classical prediction models. To the best of our knowledge, our team is the first to creatively use matrix models to combine the ARCO staging, JIC classification, and necrotic lesion area of the femoral head, allowing these three methods to complement each other and perfectly utilize their respective strengths, thereby making the prediction results more realistic and reliable.

In this study, our team listed many factors that affect femoral head necrosis and collapse, and found that older patients are more prone to collapse. Reportedly, aging rats have lower bone trabecular density and become more fragile than do young rats [22]. In addition, we listed three main factors that affect the collapse of femoral head necrosis and found that the collapse rate of ARCO II stage is higher than that of I stage. The collapse rate of necrotic lesions at the lateral column position of JIC classification C1 and C2 is much higher than that of the A and B stages of the medial column. The larger the necrotic area, the higher the collapse rate of the femoral head. The above facts confirm that these prediction methods for collapse of femoral head are still significant.

We used a matrix model score that combined the three prediction methods and found that only one hip collapsed in the femoral head with a score of 1, resulting in a very low collapse rate (4.0%). Follow-up revealed that the necrotic lesion was located on the medial side; however, the pain was severe and medication or other physical therapies were not effective. Therefore, total hip replacement surgery should be preferred when some patients have less severe imaging data; nonetheless, strong symptoms such as hip joint pain and limited mobility are also worth considering, keeping in mind the subjective feelings of the patients [23, 24]. Our study showed that the collapse rate of the femoral head with a score of 2 (32.3%) was lower than that with a score of 4 (58.8%), while the collapse rate of the femoral head with a score of 8 was as high as 90%. This indicates that as the collapse rate scores increases, the collapse rate of the femoral head also increases. A score of 2 indicates that only one of the three important influencing factors has a relatively small impact. Thus, medication or shock wave therapy can be chosen [25, 26]. The collapse rate with a score of 4 was relatively high. First, it should be given the most attention and patients should be instructed to follow-up regularly. Secondly, these patients can choose hip preservation surgery, such as osteotomy, core decompression or bone marrow derived cell therapy to prevent the progression of collapse [27–29]. The majority of patients with a score of 8 have a poor prognosis, ultimately leading to collapse. Once collapse occurs, due to the limited lifespan of the prosthesis, the patient's age should be considered when choosing therapy method [30, 31]. The previous research has found that THA with ultra-short uncemented stem or ceramic-on-ceramic bearing provides successful survival and functional outcomes in young patients, while for skeletally immature patients, individualized surgical treatment should be chosen according to the patient's own situation [32–34].

It can be seen that our proposed prediction model can effectively improve the collapse predictive ability of the three classical models and has a good guiding role in distinguishing the collapse progression of patients with no collapse at different stages. Previous retrospective studies [35] have focused on the collapse of hospitalized patients, without paying attention to the progression of femoral head necrosis collapse in outpatient patients. In this study, we followed up outpatient patients for 5 years and found that the first 3 years constituted an important stage of progress. Moreover, Wang Peng [36] pointed out that the progression of femoral head necrosis collapse is rapid, thus attention should be paid to limiting weight bearing in daily life and early intervention and treatment are needed to preserve the hip joint and delay femoral head collapse.

Our study inevitably has limitations. Firstly, the sample size was small, which may result in some prediction bias; however, we strictly followed the inclusion and exclusion criteria to collect cases, thereby making the data more authentic. Secondly, this was a single-center retrospective study; hence, the next step will be to conduct a multicenter, prospective, and more convincing study.

## Conclusion

In summary, we creatively combined the three classical prediction methods for evaluating the progression of the natural collapse course based on the matrix model and found that the higher the score of the matrix model, the higher the femoral head collapse rate, and this indicates that the collapse of the femoral head is closely related to the location, staging, and area of necrotic lesions. Specifically, the matrix model has a potential value in predicting femoral head collapse and guiding treatment selection.

## Data Availability

Due to patient privacy and confidentiality considerations, the dataset that was generated and/or analyzed as part of the present study is not publicly available, but can be obtained from the corresponding authors upon reasonable request.

## Abbreviations

NONFH: Non-traumatic osteonecrosis of the femoral head
ARCO: The Association Research Circulation Osseous
JIC: Japanese Investigation Committee
OR: Odds ratio
CI: Confidence interval
AUC: Area under the curve
